# Tetrapyrroles as Endogenous TSPO Ligands in Eukaryotes and Prokaryotes: Comparisons with Synthetic Ligands

**DOI:** 10.3390/ijms17060880

**Published:** 2016-06-04

**Authors:** Leo Veenman, Alex Vainshtein, Nasra Yasin, Maya Azrad, Moshe Gavish

**Affiliations:** Department of Neuroscience, Faculty of Medicine, Rappaport Family Institute for Research in the Medical Sciences, Technion-Israel Institute of Technology, Ephron Street, P.O.B. 9649, Bat-Galim, Haifa 31096, Israel; alexanderv21184@gmail.com (A.V.); blackpearl.black2@gmail.com (N.Y.); mayabz@gmail.com (M.A.)

**Keywords:** TSPO, tetrapyrrole, eukaryotes, prokaryotes, TSPO ligand binding, TSPO binding site structures, cell function, stress, homeostasis, life expectancy

## Abstract

The 18 kDa translocator protein (TSPO) is highly 0conserved in eukaryotes and prokaryotes. Since its discovery in 1977, numerous studies established the TSPO’s importance for life essential functions. For these studies, synthetic TSPO ligands typically are applied. Tetrapyrroles present endogenous ligands for the TSPO. Tetrapyrroles are also evolutionarily conserved and regulate multiple functions. TSPO and tetrapyrroles regulate each other. In animals TSPO-tetrapyrrole interactions range from effects on embryonic development to metabolism, programmed cell death, response to stress, injury and disease, and even to life span extension. In animals TSPOs are primarily located in mitochondria. In plants TSPOs are also present in plastids, the nuclear fraction, the endoplasmic reticulum, and Golgi stacks. This may contribute to translocation of tetrapyrrole intermediates across organelles’ membranes. As in animals, plant TSPO binds heme and protoporphyrin IX. TSPO-tetrapyrrole interactions in plants appear to relate to development as well as stress conditions, including salt tolerance, abscisic acid-induced stress, reactive oxygen species homeostasis, and finally cell death regulation. In bacteria, TSPO is important for switching from aerobic to anaerobic metabolism, including the regulation of photosynthesis. As in mitochondria, in bacteria TSPO is located in the outer membrane. TSPO-tetrapyrrole interactions may be part of the establishment of the bacterial-eukaryote relationships, *i.e.*, mitochondrial-eukaryote and plastid-plant endosymbiotic relationships.

## 1. Concise Introduction to the Theme

**Figure 1:**
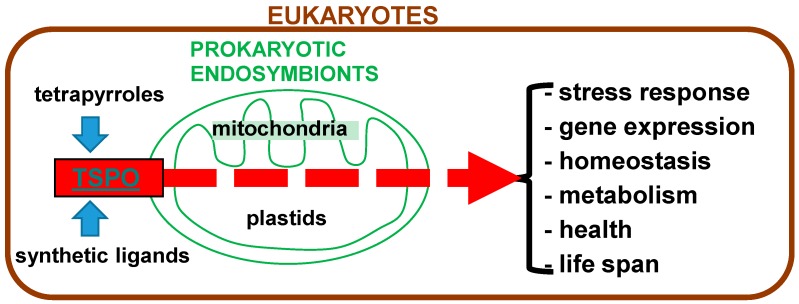
Overview of the subject of this review. Endogenous ligands (tetrapyrroles) as well as synthetic ligands for translocator protein (TSPO) affect functions of free living prokaryotes as well as the derived endosymbionts present as mitochondria and plastids in eukaryotes. A few of these functions modulated by TSPO and its ligands are listed on the right-hand side.

Interactions between the 18 kDa translocator protein (TSPO) and tetrapyrroles, including the tetrapyrrole protoporphyrin IX (PPIX), have been studied for several decades, in various species. These species cover animals, plants, fungi, bacteria, and archea, *i.e.*, eukaryotes as well as prokaryotes. TSPOs as well as tetrapyrroles are known to be involved in various vital functions regarding molecular cell biology as well as organismal functions (Figure 1). Thus, it is worthwhile to present an overview of the knowledge that has been gathered so far, and mention the future perspectives for research regarding interactions between TSPO and tetrapyrroles. In the end such research will reveal further potential implications regarding health and disease, and even plant cultivation. In this review, apart from interactions between TSPO and tetrapyrroles, including PPIX as an endogenous ligand, the effects of synthetic TSPO ligands in the context of TSPO-tetrapyrrole interactions are also presented (summarily indicated in Figure 1).

## 2. The 18 kDa Translocator Protein (TSPO)

### 2.1. General TSPO Characteristics and Functions

The 18 kDa translocator protein (TSPO) is a highly conserved protein in eukaryotic as well as prokaryotic species and is involved in various life essential functions (Table 1 and Table 2) [1,2]. Previously, TSPO was known as peripheral-type benzodiazepine receptor (PBR) because of its ability to bind benzodiazepines in various peripheral tissues in mammals [1,3,4]. A major intracellular location of this five-α-helices membrane-spanning protein in eukaryotes is the outer mitochondrial membrane at the contact sites with the inner mitochondrial membrane [4,5,6]. TSPO can form a complex with the voltage-dependent anion channel (VDAC, 32 kDa) and the adenine nucleotide translocator (ANT, 30 kDa), which are located at the outer and inner mitochondrial membrane, respectively [1,6]). The isoquinoline carboxamide PK 11195, the benzodiazepine Ro5-4864, and the indole derivative FGIN-1-27 are the classical synthetic ligands of TSPO [1,7]. Their full names are, respectively, 1-(2-chlorophenyl)-*N*-methyl-*N*-(1-methylpropyl)-3-isoquinoline carboxamide; 7-chloro-5-(4-chlorophenyl)-1-methyl-3*H*-1,4-benzodiazepin-2-one; and *N*,*N*-di-n-hexyl 2-(4-fluorophenyl)indole-3-acetamide.

A well-known endogenous TSPO ligand is the tetrapyrrole protoporphyrin IX (PPIX) [8,9]. The present review deals with the potential of tetrapyrroles to modulate TSPO functions in animals, plants, and bacteria. It has been found that TSPO has various functions in mammals, such as programmed cell death induction, regulation of mitochondrial membrane potential transition (MPT) including mitochondrial membrane potential (ΔΨm) collapse, respiratory chain regulation, cholesterol transportation, regulation of steroidogenesis, heme metabolism (heme is PPIX containing a Fe^2^⁺ ion in its center), anion transportation, modulation of voltage-dependent anion channel (VDAC) opening, immune response, glial activation related to brain damage, cell growth and differentiation, and cancer cell proliferation [1,4,10,11,12,13,14] (see also Table 2). At organismal levels, the effects include modulation of endocrine, reproductive, and cardiovascular functions, local responses to brain damage due to injury and disease, and other neuropathological, emotional, and mental disorders, in particular including responses to stress [1,12,15,16,17,18,19,20,21,22], as well as life span enhancement [19,21,22] (see also Table 2). At cellular levels, programmed cell death regulation by the TSPO may include changes in TSPO expression levels, and typically includes mitochondrial reactive oxygen species (ROS) generation, cardiolipin oxidation, and collapse of the ΔΨm, all under the control of the TSPO and its ligands [10,14,23,24,25,26,27] (see also Table 2). Previous studies have shown that knockdown of TSPO by genetic manipulation as well as application of its synthetic ligands Ro5-4864, PK 11195, and FGIN-1-27 can protect various human and animal cell lines against apoptotic cell death [14,18,24,26,28]. Indicated in Table 2, TSPO-associated functions studied in plants and bacteria are reminiscent of several TSPO-associated functions in animals. This is also discussed in Section 6, Section 7 and Section 8 of this review, respectively dealing with: “Bacteria, TSPO and tetrapyrroles”; “Plants, TSPO and tetrapyrroles”; and “TSPO-tetrapyrrole interactions from an evolutionary perspective”.

Also, TSPO involvement in steroid production, including neurosteroids, has attracted a lot of attention [18,29,30,31] (see also Table 2). These studies emphasize that aberrant regulation of steroid production via TSPO activity can be linked to cancer, neurodegeneration, neuropsychiatric disorders, and primary hypogonadism. TSPO ligands have been proposed as therapeutic agents to regulate steroid levels in the brain and reproductive system. *Vice versa*, it is also well known that at system levels, various types of steroids modulate TSPO expression [1,15,18]. It is also becoming more and more appreciated that TSPO is involved in the differentiation of various cell types [22,32,33] (see also Table 2). Synthetic TSPO ligands promoting neuronal differentiation have been proposed as therapeutic agents for the repair of brain and spinal cord damage due to injury and disease [21]. Recent studies have also revealed the involvement of TSPO in the modulation of nuclear gene expression, giving some explanation as to how TSPO can be involved in so many and such diverse functions [34,35,36] (see also Table 2). As this regulation of gene expression most likely takes place via the retrograde mitochondrial-nuclear signaling pathway for the regulation of nuclear gene expression, it probably is a TSPO function that can be found in all eukaryotes.

### 2.2. TSPO Ligands, Endogenous and Synthetic

Several endogenous TSPO ligands have been identified. PPIX, which was first reported in 1987 as a TSPO ligand [8,37], is the most studied tetrapyrrole in this respect [9]. Other endogenous TSPO ligands include: phospholipase A2 (PLA2) (*Naja naja*) [38], and diazepam binding inhibitor (DBI) and its post-translational products [39,40,41,42]. Apart from PPIX no other small endogenous molecules are known that display ligands binding to the TSPO in animals. It would be worthwhile to endeavor targeted research to detect small endogenous molecules binding specifically to TSPO in animal species, a phenomenon as, for example, suggested by plant TSPO research (see Section 7 dealing with: “Plants, TSPO and tetrapyrroles”).

**Figure 2:**
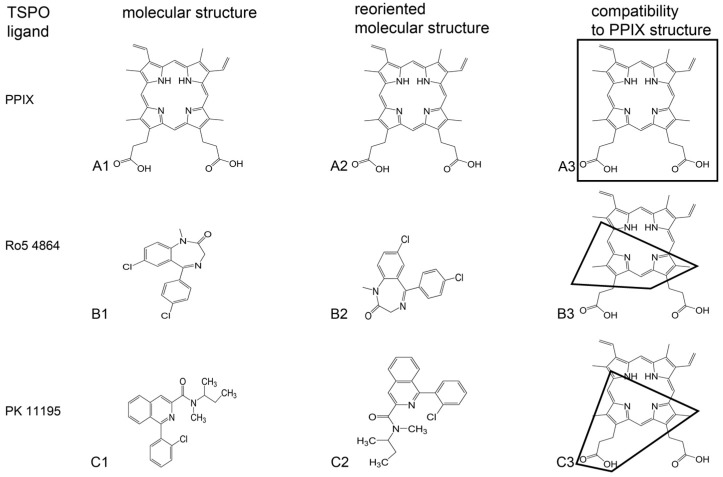

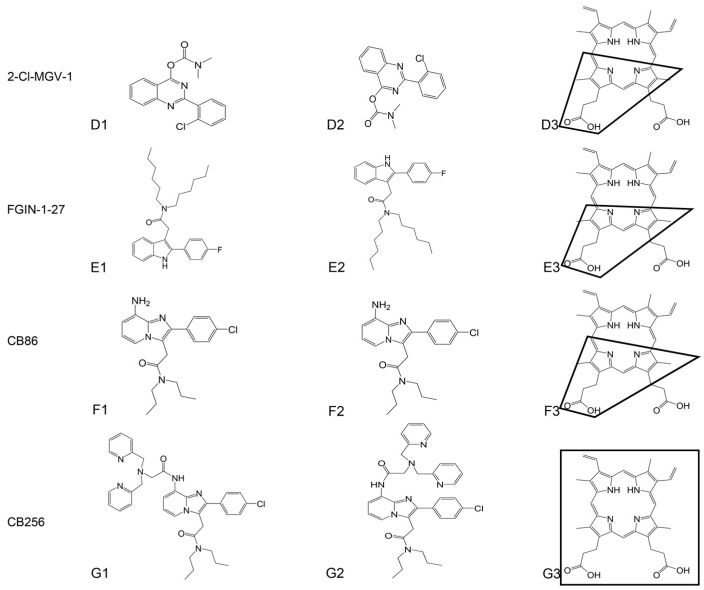
This figure presents line drawings of the molecular structures of seven known TSPO ligands (listed in the most left hand column), to visualize for each one the structural compatibilities of the synthetic ligands with the molecular structure of other synthetic ligands and the endogenous TSPO ligand PPIX. For orientation in the figure, adjacent to each molecular structure the letter refers to the row (lined up with the compound's name) and the numbers refer to the columns related to the molecular structure characteristics (*i.e.*, 1 relates to Molecular Structure, 2 relates to Reoriented Molecular Structure, and 3 relates to Compatibility to PPIX structure). These TSPO ligands were first described as such by: Verma *et al.* [8] (PPIX), Le Fur *et al.* [44] (Ro5-4864 and PK 11195), Vainshtein *et al.* [22] (2-Cl-MGV-1), Romeo *et al.* [45] (FGIN-1-27), Denora *et al.* [46] (CB86 and CB256). Their full names at the left hand beginnings or their rows are, respectively : 3-[18-(2-carboxyethyl)-8,13-bis(ethenyl)-3,7,12,17-tetramethyl-22,23-dihydroporphyrin-2-yl]propanoic acid (protoporphyrin IX ; abbreviation PPIX in row A), 7-chloro-5-(4-chlorophenyl)-1-methyl-3*H*-1,4-benzodiazepin-2-one (Ro5-4864 in row B); 1-(2-chlorophenyl)-*N*-methyl-*N*-(1-methylpropyl)-3-isoquinoline carboxamide (PK 11195 in row C); [2-(2-chlorophenyl)quinazolin-4-yl dimethylcarbamate] (2-Cl-MGV-1 in row D); *N*,*N*-di-n-hexyl 2-(4-fluorophenyl)indole-3-acetamide (FGIN-1-27 in row E); 2-(8-amino-2-(4-chlorophenyl)*H*-imidazo[1,2-*a*]pyridin-3-yl)-*N,N*-dipropylacetamide (CB86 in row F) 2-(8-(2-(bis(pyridin-2-yl)methyl)amino)acetamido)-2-(4-chlorophenyl)*H*-imidazo[1,2-*a*]pyridin-3-yl)-*N*,*N*-dipropylacetamide (CB256 in row G). In the first, left hand column the names of the ligands are given as they are generally used in the scientific community (not numbered here). In the second column (indicated with #1) the molecular structures are given as they are typically presented in the literature. In the third column (indicated with #2) the molecular structures are reoriented to facilitate visualization of a potential match with a corresponding part of PPIX. This reorientation typically is no more than flipping and rotating the original drawing, if required at all. Regarding the drawing of “CB256”, rotations of several bonds are applied (using ChemBioDraw™) to achieve a configuration that matches the structure of PPIX. In the fourth, most right handed column (indicated with #3), in each row, the PPIX molecular structure is presented. In this fourth column, for each row, angular shapes are drafted, outlining the parts of PPIX that may potentially correspond to the full molecular structures of the ligands in the rows in question. Thus, this figure presents structural characteristics common to various TSPO ligands. One can assume that the structural commonalities are related to shared functions (as well as affinity for the TSPO), while the structural differences may be related to differences in effects (as well as differences in affinity for the TSPO). The molecular structures were drawn with the aid of ChemBioDraw ™ of PerkinElmer, 940 Winter Street, Waltham, MA, USA.

Since the discovery of TSPO in rats by Braestrup and Squires [3], an increasing number of synthetic TSPO ligands are incessantly being developed [7,21,27,43]. As mentioned, PK 11195, Ro5-4864, and FGIN-1-27 can be considered classical TSPO ligands [44,45] (presented in Figure 2). Their full names are, respectively, 1-(2-chlorophenyl)-*N*-methyl-(1-methylpropyl)-3 isoquinolinecarboxamide; 7-chloro-5-(4-chlorophenyl)-1-methyl-3*H*-1,4-benzodiazepin-2-one; and *N*,*N*-di-n-hexyl 2-(4-fluorophenyl)indole-3-acetamide. Later synthetic TSPO ligands encompass: (i) derivatives of Ro5-4864; (ii) derivatives of the 2 aryl-3-indoleacetamide FGIN-1, including FGIN-1-27; and several other types of molecules [7,21,27,43]. In general, the most common structure of a synthetic TSPO ligand includes a backbone of three carbocycles, typically including heteroatoms such as O or N [7,21,27,43,44,45]. In addition, carbon-based side chains including an acetamide component are frequently part of these TSPO ligands. Furthermore, halogenations at several locations, and/or additional carbocycles linked to the basic structure, are often part of the synthetic TSPO ligands. A few examples of synthetic TSPO ligands, together with the endogenous TSPO ligand PPIX are given in Figure 2. Apart from these small molecules, based on carbocycles, synthetic peptides with the motif STXXXXP can also act as TSPO ligands.

Comparisons of effects and structures of endogenous ligands, such as PPIX, with those of synthetic ligands (as illustrated by Figure 2) may lead to insights into the functional aspects of their interactions with the TSPO. This may include their potential structural interactions with ligand binding sites present on the TSPO. In turn, these elucidations may inform us what is essential for the design of efficacious TSPO ligands, for example for treatment of diseases while avoiding undesired concomitant side effects. As the scope of functional capabilities of the TSPO is broad (Table 2), we are dealing with a double-edged sword. While specific TSPO ligands may display beneficial characteristics regarding various diseases, at the same time such ligands targeting a specific disease may induce effects not related to the disease in question, and may even show adverse additional effects. Knowledge of endogenous ligands may teach us which aspects, affinity-wise and functional, one should focus on for the development of TSPO ligands targeting specific diseases.

## 3. Tetrapyrroles, including Protoporphyrins such as PPIX, as Ligands for TSPO

### 3.1. Tetrapyrroles Binding to TSPO

Interestingly, as TSPO is an evolutionarily conserved protein in archea, bacteria, fungi, plants, and animals (Table 1), found throughout all tissues studied [1,2,21], tetrapyrroles, in a similar vein, are probably one of the most ancient prosthetic groups in all kingdoms of living organisms and comprise the most abundant pigment molecules on earth [47]. The multifunctionality of tetrapyrroles, in particular in association with their interactions with TSPO in eukaryotes as well as prokaryotes in this respect, suggests a relationship between TSPO and tetrapyrroles, at least from the geological time point that endosymbiotic relationships between specific bacterial species and eukaryotes were established (see also Section 8 dealing with: “TSPO-tetrapyrrole interactions from an evolutionary perspective”). Tetrapyrroles are a class of chemical compounds that contain four pyrrole rings held together by one-carbon bridges (=(CH)– or –CH: two units) or by direct covalent bonds, in either a linear or a cyclic fashion. Tetrapyrroles are involved in metabolism in all kingdoms of living organisms. In the animal kingdom, well-known tetrapyrroles are porphyrins that are part of the heme synthesis pathway [48]. In plants, tetrapyrroles are part of the pathways leading to heme formation and chlorophyll [49]. In cyanobacteria and red algae, such pathways lead to the formation of different phycobilines [49]. Below, we will give an overview of what is known thus far regarding interactions between tetrapyrroles and TSPO.

In 1987, it was reported that porphyrins (cyclic tetrapyrroles with prominent physiological functions), extracted from rat as well as human tissue, in particular PPIX (Figure 2), present themselves as endogenous ligands for TSPO [8,37]. In this respect, the endogenous PPIX and the synthetic PK 11195 showed relative constancy in affinity (in the nM range). In contrast, the affinity of the synthetic benzodiazepine Ro5-4864 was shown to vary several orders of magnitude in competing for receptors in different organs and species [8,37,50,51]. One typical aspect of the Ro5-4864 molecule is that it lacks an elongated side chain (Figure 2). In contrast, both PK 11195 and PPIX display elongated side chains containing a number of carbon atoms (Figure 2). Based on present knowledge, one can suggest that the side chain presenting a number of carbon atoms attached to the part of the molecule binding to the TSPO in question is important for constancy in affinity [22]. In Table 3, TSPO functions are listed that were found to be modulated by tetrapyrroles in animals, plant, and bacteria, as presented in this review.

### 3.2. Implications of Tetrapyrrole-TSPO Interactions

It was suggested that ligands specific to TSPO could be applied for the treatment of porphyrias [52]. Porphyrias are diseases in which porphyrins ccumulate [53]. Hepatic protoporphyria induced by DDC (3,5-diethoxycarbonyl-1,4-dihydrocollidine) includes increased levels of PPIX and *N*-methylprotoporphyrin IX (N-MePPIX), which is associated with a decrease in TSPO ligand binding in the liver, as measured in rats’ liver homogenates [54]. This included decreased affinity for Ro5-4864 and PK 11195, as well as a 55% decrease in the maximum number of binding sites (Bmax). Further studies on cultured hepatocytes suggested that, depending on the energetic states of the mitochondria, TSPO-PPIX interactions may have multifunctional effects, including membrane permeability transition (MPT) and transport of PPIX across the mitochondrial membranes [55]. In this study [55], in hepatocyte cultures de-energized by rotenone, nanomolar concentrations of PPIX potentiated the induction of the MPT, *i.e.*, induced ΔΨm collapse, and enhanced the extent of cell killing. In short, PPIX enhanced the effects of rotenone. This appears to be the opposite of what happens with nanomolar concentrations of PK 11195, Ro5-4864, and FGIN-1-27 in U118MG cells, which at these concentrations counter ΔΨm collapse and cell death otherwise induced by ammonia [28]. This suggests that at least in this respect, these synthetic TSPO ligands can counter the functions of the endogenous PPIX. As a specific example, PK 11195 as well as TSPO knockdown by siRNA can counteract the cytotoxic effects of hemin (PPIX containing a ferric iron ion with a chloride ligand) in colonic epithelial (Caco-2) cells [56]. Already early on it was found that the TSPO ligands PK 11195, Ro5-4864, and PPIX had different functional effects. For example, Ro5-4864 and PK 11195 could modulate prolactin-stimulated mitogenesis in the Nb2 lymphoma cell, while PPIX had no such effect [57]. Rats suffering from porphyria induced by DDC showed interesting effects of PK 11195 administration (15 mg/kg/day) [58]. This PK 11195 administration aggravates PPIX accumulation and cellular damage in the liver. It was suggested that in this paradigm, PK 11195 blocks the binding of PPIX to TSPO, thereby elevating the content of PPIX in the liver [59], *i.e.*, it then already was assumed that TSPO contributes to metabolization of PPIX, which was corroborated by later studies [13].

In rat liver mitochondria, PK 11195 and N-MePPIX dose-dependently stimulate cholesterol translocation and incorporation into inner membranes, while PPIX and Ro5-4864 are ineffective in this respect [59]. In another study, time- and dose-dependent effects of synthetic and endogenous TSPO ligands were seen. Briefly, PK 11195, N-MePPIX, and PPIX either stimulated mitochondrial 27-hydroxylation of [4-14C] cholesterol *in vitro* (PK 11195 and N-MePPIX being more effective than PPIX), or at relatively long-time exposures and increased doses of these TSPO ligands, mitochondrial 27-hydroxylation of [4-14C] cholesterol was decreased [60]. Thus, bimodal effects on the acidic pathway to bile acids, depending on the concentrations of these endogenous and synthetic TSPO ligands and exposure times, were reported. In contrast to PK 11195, N-MePPIX, and PPIX, mentioned effects were not seen for Ro5-4864 and hemin [61]. Various studies have suggested that the concentration-dependent bimodal effects of TSPO ligands may be associated with the presence of high affinity and low affinity binding sites [10,28,60,62]. For example, bimodal effects of TSPO ligands on ammonia-induced toxicity showed that nanomolar concentrations of PK 11195, Ro5-4864, and FGIN-1-27 protected U118MG cells from ammonia-induced cell death while these same TSPO ligands applied at µM concentrations enhanced ammonia-induced cell death of U118MG cells [28].

In human tissues and cells, applying quantitative evaluation of the positron emission tomography (PET) signal of the ^11^C-PBR28 TSPO ligand revealed high affinity binding sites (HAB) and low affinity binding sites (LAB) [63]. In particular, PBR28 can bind the TSPO with high affinity (binding affinity as indicated by the dissociation constant *K*_i_ ~4 nM), low affinity (*K*_i_, ~200 nM), or mixed affinity (two sites with *K*_i_, ~4 and ~300 nM). Other TSPO ligands, including DAA1106, DPA713, PBR06, PBR111, and XBD173 also bind with different affinities to TSPO binding sites. However, PK 11195 does not present such a distinction in affinity [63,64]. The differences in affinity appear to relate to two types of human TSPOs differing at just one amino acid site. In this common polymorphism, an alanine at position 147 of the wild-type TSPO is replaced by threonine [65]. An 18 kDa translocator protein (TSPO) polymorphism explains differences in binding affinity of the PET radioligand PBR28. It has been suggested that in the cases of PBR28 and other second-generation ligands, A147T mTSPO might no longer be able to retain the same structural and dynamic profile as the wild-type protein and thus binds these ligands with lower affinity [66]. Nonetheless, this polymorphism does not affect affinity for PK 11195 [67].

Regarding interactions of synthetic TSPO ligands with functions of the endogenous TSPO ligand PPIX, in one paradigm the effects of PK 11195 and Ro5-4864 on the metabolism and function of PPIX were studied. For this study, accumulation of photoactive PPIX was achieved by application of the exogenous PPIX precursor δ-aminolaevulinic acid (ALA) to rat pancreatoma AR4-2J cells in culture [68]. Under these conditions, exposure to light (λ > 400 nm) at an intensity of 0.2 mW·cm^2^ for 8 min resulted in cytolysis. PPIX generates singlet oxygen upon illumination, *i.e.*, it generates ROS. PK 11195 and Ro5-4864 (at 10 and 20 µM for both ligands) exerted a photoprotective effect in this paradigm [68]. In another study it was found that TSPO can serve to reduce PPIX levels, most likely in association with ROS generation, as determined with TSPO knockdown [13]. A similar effect was induced by 25 µM of PK 11195 [13]. The suggestion that TSPO can serve to catalytically metabolize PPIX to tetrapyrrole products other than hemin in human U118MG glioblastoma cells [13] was recently corroborated for TSPO from *Bacillus cereus*, *Xenopus*, and mammals, in addition to humans [69,70]. Thus, one type of interaction between PPIX and TSPO may simply serve to catabolize excess PPIX in conjugation with ROS generation [13].

Thus, it was found that interactions between TSPO and PPIX cover various aspects, ranging from receptor ligand interactions, modulation of the mitochondrial MPT, and even including collapse of the ΔΨm, ROS generation, initiation of programmed cell death, gene expression regulation, cholesterol transport, and heme transport and metabolism. From these studies it can be concluded that it is worthwhile to apply further studies to deepen our insights into TSPO-PPIX interactions. For example, applying recombinant mouse TSPO expressed in *Escherichia coli* showed that PPIX could displace PK 11195 binding in *E. coli* expressing this recombinant mouse gene product [71]. Moreover, induced TSPO protein expression in *E. coli* protoplasts caused an uptake of PPIX that could be completely inhibited by cholesterol and, to a lesser extent, inhibited by PK 11195 and Ro5-4864 [71]. In another study it was found that enhanced TSPO levels in glioma cells were associated with enhanced PPIX production [72]. Also, in rat *in vivo*, PPIX binding to the TSPO could be demonstrated with positron emission tomography, giving further indication of TSPO-tetrapyrrole interactions [73].

### 3.3. Implications of Tetrapyrrole-TSPO Interactions for Brain Disease

Focusing on TSPO-tetrapyrrole interactions, in a model for acute hepatic encephalopathy, ammonia-induced astrocyte swelling in culture could be attenuated by PK 11195 and PPIX, while Ro5-4864, diazepam binding inhibitor (DBI51-70), and octadecaneuropeptide exacerbated the swelling [74]. To gain a better understanding of PPIX-TSPO interactions in relation to inflammatory processes in the brain, effects on free radical generation by TSPO ligands were studied in cultured neural cells, including primary cultures of rat brain astrocytes and neurons as well as cells of the murine BV-2 microglial cell line [75]. For this purpose, the fluorescent dye dichlorofluorescein-diacetate was used. Free radical production was measured at the time points of 2, 30, 60, and 120 min of treatment with the TSPO ligands PK 11195, Ro5-4864, and PPIX (all at 10 nM). In astrocytes, all ligands showed a significant increase in free radical production at 2 min. The increase was short-lived with PK 11195, whereas with Ro5-4864 it persisted for at least 2 h. PPIX caused an increase at 2 and 30 min, but not at 2 h. Similar results were observed in microglial cells [75]. In this same study, the application of PK 11195 and PPIX to neurons showed an increase in free radical production only at 2 min, while Ro5-4864 had no effect. All in all, even though differences from cell type to cell type and ligand to ligand can be discerned, TSPO ligands can induce free radical production virtually instantly when applied to cells. Cyclosporin A (CsA), an inhibitor of the MPT, could prevent free radical formation by these TSPO ligands. CsA (1 µM) completely blocked free radical production following PK 11195 and Ro5-4864 treatment in all the cell types of this study [75]. CsA was also effective in blocking free radical production in astrocytes following PPIX treatment, but it failed to do so in neurons and microglia. These studies indicated that exposure of neural cells to TSPO ligands generates free radicals, and that the MPT may be involved in this process [75]. Again, the study by the group of Norenberg [74,75] makes it clear that different TSPO ligands have different effects. Furthermore, the effects of the TSPO ligands were different from cell type to cell type.

Later studies applying various agents typically inducing programmed cell death showed that ROS generation at mitochondrial levels, measured with acridine orange 10-nonyl bromide (NAO) in U118MG cells, could be attenuated by TSPO knockdown, as well as by the TSPO ligands PK 11195, Ro5-4864, and FGIN-1-27 (applied for 24 h, optimal concentrations to achieve these effects typically range around 25 and 50 µM) [14,26,28]. Studies such as these substantiated that TSPO serves to initiate programmed cell death, including ROS generation, MPT, collapse of the ΔΨm, and mitochondrial cytochrome C release [11,27,28]. Such ROS generation and MPT can also be part of the retrograde mitochondrial nuclear signaling expression pathway for regulation of nuclear gene expression [34,35,36]. An important aspect of these studies discussed above is that TSPO ligands, including PPIX, modulate ROS generation. This modulation of ROS generation typically is bimodal. In particular, with short durations of TSPO ligand exposures as well as with the application of low concentrations of ligands, ROS generation is enhanced. This is in contrast with the reduction of ROS generation induced by long durations of TSPO ligand exposures as well as by the application of high concentrations of TSPO ligands. This gives some indication as to why and how TSPO ligands, in a time- and dose-dependent way, can have opposite functional effects, *i.e.*, reductions and enhancements of ROS generation may serve as signals to induce particular functions, and may even be integral to such functional effects. Thus, while each effect in its own paradigm is very reproducible and TSPO-dependent, there is very high, context-associated variability in response, including the effects of the duration of exposure to TSPO ligands, the concentration of the TSPO ligands, and the cell types used. Importantly, PPIX is not an exception regarding these phenomena. These time-dependent and dose-dependent effects of TSPO ligands may reflect the TSPO’s function to maintain homeostasis of various cell types, and moreover the health of the complete organism, including the brain in animals [7,76,77].

### 3.4. Tetrapyrrole-TSPO Interactions in Insects

Apart from vertebrates, TSPO is also found in insects [2]. A few studies regarding TSPO in association with tetrapyrroles have been performed in insects. First of all, the *Drosophila* homolog for TSPO, CG2789/dTSPO, has been identified [19]. This dTSPO was then inactivated by P-element insertion, RNAi knockdown, and inhibition by ligands (PK 11195 and Ro5-4864). Such inhibition of dTSPO in turn inhibited wing disk apoptosis in response to γ-irradiation or H_2_O_2_ exposure [19]. In the whole animal, dTSPO inhibition enhanced the male fly life span and inhibited Aβ42-induced neurodegeneration. These effects found in insects [19] are reminiscent of the control of TSPO on apoptosis, life span, and neurodegeneration in mammals [11,21,78]. Interestingly, PPIX can also enhance the lifespan of *Drosophila*, both female and male [79,80]. Data regarding *Drosophila* reported by Curtis *et al.* [81] suggest that upregulation of the mitochondrial antioxidant manganese superoxide dismutase (Mn-SOD) and a retrograde signal of ROS from the mitochondria can serve as intermediate steps in life span extension of *Drosophila*. PPIX, PK 11195, and Ro5-4864 were all found to enhance mitochondrial processing of the hMn-SOD precursor protein, suggesting a role for the TSPO in the regulation of mitochondrial transport of proteins, as well as life span extension [82]. It was also found in *Drosophila* that TSPO serves to counteract infection and promote wound healing [83]. In addition, PPIX can reduce genetic damage in *Drosophila* [81]. In embryonic grasshoppers, PPIX promotes the migration of neurons [84]. In adult mosquitoes, PPIX inhibits heme metabolism [85]. It appears that *Drosophila* presents an interesting model to study TSPO function in a whole animal in association with PPIX effects, in particular regarding health issues.

## 4. Functional Effects of PPIX and Synthetic TSPO Ligands in Mammals, including Human

### 4.1. Differences between PPIX and Synthetic Ligands in Interactions with TSPO

Studies regarding TSPO-PPIX interactions have also been applied to human primary cells [86,87,88]. Moreover, these studies have been done in comparison to synthetic TSPO ligands. First of all, it was shown that TSPO is abundant in primary human osteoblasts in cell culture showing ligand affinity in the nM range as assayed with [^3^H]PK 11195 [86]. In primary human osteoblasts in culture, following exposure to PPIX (10 µM), cellular [^18^F]-FDG incorporation, mitochondrial mass, and ATP content were suppressed, indicative of reduced metabolism [87]. Cellular proliferation was not affected. The ΔΨm collapsed, *i.e.*, MPT was enhanced, while no increase in apoptotic cell death was observed. Nonetheless, lactate dehydrogenase activity, indicative of overall cell death, was enhanced in culture media. Accordingly, cell numbers decreased. Protein expression of TSPO, VDAC1, and hexokinase 2 decreased [87]. It was found that the TSPO ligands PK 11195, Ro5-4864, and FGIN-1-27 applied at a 10 µM concentration did not exert the exact same effects as PPIX in the primary osteoblast cell culture [86,87,88]. For example, PK 11195 did not significantly affect cell death, maturation, [^18^F]-FDG incorporation, and hexokinase 2 protein expression. There was an increase in mitochondrial mass and mitochondrial ATP content, and a reduction in ΔΨm collapse, *i.e.*, a decrease of MPT. The differences and similarities are discussed in more detail in a recent study submitted for publication [89]. It is considered that the functional differences may due to the structural differences between PPIX, Ro5-4864, PK 11195, and FGIN-1-27 (Figure 2) [89].

By studies on homogenates *in vitro*, as well as on cell culture and animals *in situ*, we have investigated which molecular parts are important for functional effects of tricyclic TSPO ligands based on the quinazoline scaffold [21,22,90,91]. One of these tricyclic TSPO ligands based on the quinazoline scaffold, named 2-Cl-MGV-1 [2-(2-chlorophenyl)quinazolin-4-yl dimethylcarbamate], protected cells of astroglial origin from glutamate-induced cell death, induced differentiation of neuronal progenitor cells, ameliorated behavioral abnormalities of R6-2 mice (a transgenic mouse model for Huntington Disease), and enhanced the life span of R6-2 mice. The ligand 2-Cl-MGV-1 is given as one example for structural characteristics of TSPO ligands in Figure 2. These studies have indicated that the side chains of TSPO ligands affect affinity [22]. The N atoms in the central carbocycles (the second carbocycle of the three carbocycles) affect affinity as well as function, *i.e.*, regulation of programmed cell death. This, for example, has been determined by comparisons of derivatives of phthalazines, quinoxalines, and quinazolines [90]. As 2-Cl-MGV-1 and MGV-1 (2-phenylquinazolin-4-yl dimethylcarbamate) better protect against cell death induction than PK 11195, and also are more effective in the induction of cell differentiation than PK 11195 [21,22], it appears that the location of an N atom on the side of the central carbocycle facing away from the side chain is elementary for proper functioning. This location of N atoms can be also recognized in PPIX (Figure 2), *i.e.*, the N atoms surrounding the center of the PPIX molecule that face away from the side chains at the outside of the cyclic-shaped PPIX. The N atoms at the center of porphyrins are well known to be essential for function, including the holding of metals. Halogenation by a single halogen of the third, rotatable carbocycle in tricyclic TSPO ligands based on a quinazoline scaffold affects function not affinity. In particular, this halogenation prevents cell death induction at higher concentrations in contrast to the typical lethal effects of “normal” TSPO ligands at such concentrations [10,22,26]. Further enhancement of halogenation, from one to more halogens at this part of the compound, reduces affinity in addition to enhanced functional beneficial effects on programmed cell death [85]. This could imply that modifications of cyclic tetrapyrroles that can interact with TSPO could have beneficial effects, for example by halogenation at specific locations and/or by variations of the length and structure of the side chains. A potentially illustrative example of this in Figure 2 is CB256 ((2-(8-(2-(bis(pyridin-2-yl)methyl)amino)acetamido)-2-(4-chlorophenyl) *H*-imidazo[1,2-*a*]pyridin-3-yl)-*N,N*-dipropylacetamide). CB256 is a synthetic TSPO ligand based on a tricyclic backbone, with the addition of a side chain presenting two additional carbocycles with heteroatoms (Ns) [46]. One could consider it as a hybrid form of a tricyclic synthetic TSPO ligand and a tetracyclic TSPO ligand (reminiscent of a tetrapyrrole). *Vice versa*, one could also test whether selected segments of the tetrapyrroles by themselves could function as TSPO ligands.

### 4.2. Effects of Synthetic TSPO Ligands and the Endogenous TSPO Ligand PPIX in Blood Cells

In addition to human primary osteoblasts, TSPO-specific binding by PK 11195 and PPIX was also found in human mononuclear cells drawn from the blood circulation, a first indication that TSPO may be important for the host defense system [7,92]. Displacement studies applying [^3^H]PK 11195 indicated that TSPO ligand binding sites recognized by PK 11195 in human lymphocytes are also recognized by Ro5-4864 and endogenous ligands, including PPIX and diazepam binding inhibitor (DBI) [93]. PPIX appears to be specifically targeting mitochondrial TSPO in these cells [93]. Interestingly, while TSPO is an outer mitochondrial membrane protein, Ro5-4864 and PPIX at nanomolar concentrations do inhibit the activity of inner membrane ion channels, namely the multiple conductance channel (MCC) and the mitochondrial centum-picosiemen (mCtS) [94]. PK 11195 inhibits mCtS activity at similar concentrations. Higher concentrations of PPIX induced MCC activity [94], a first indication that TSPO ligands including the endogenous TSPO ligand PPIX can have concentration-dependent bimodal effects, as also found later for protection against and induction of programmed cell death [27]. In addition to the regulation of transport of ions and small molecules over the mitochondrial membranes, the TSPO also appears to regulate the import of proteins into the mitochondria, including the matrix [82]. For example, apart from its function as a channel for ions, MCC can serve as the pore of the import complex present in the inner mitochondrial membrane [95]. In short, MCC is also considered to be a protein import channel. One can ask the question: by which mechanisms can TSPO regulate functions of interacting outer membrane proteins, including specific protein complexes? The question then still remains: by which mechanism can TSPO regulate functions of proteins located at the inner mitochondrial membrane?

In mouse erythroleukemia (MEL) cells it was found that TSPO may be involved in cell differentiation and heme biosynthesis [96]. In this study, RNA blot analysis revealed that treatment of MEL cells with dimethyl sulfoxide to induce differentiation led to an increase in TSPO mRNA levels for up to 72 h, with a concomitant induction of mRNAs for the heme biosynthetic enzymes, coproporphyrinogen oxidase, and ferrochelatase, *i.e.*, modulation of their gene expression. These results suggested that TSPO may be involved in porphyrin transport and may even be a critical factor in erythroid-specific induction of heme biosynthesis. Later studies in human glioblastoma cells suggested that TSPO may be involved in the degradation of excess levels of PPIX, by means of ROS generation [13]. PPIX was also found to induce erythroid differentiation of the human leukemia cell line K562, which was dose-dependently inhibited by PK 11195 [97]. Exposing platelets to PPIX (10 µM) caused a significant decrease in affinity for TSPO ligand binding, whereas the number of TSPO ligand binding sites remained unaltered [98]. Thus, PPIX can interact with TSPO in all types of human blood cells [7]. Studies in other human cells have shown that TSPO is a major participant in the modulation of gene expression in human glioblastoma cells [34,35,36].

## 5. Structural Relationships between Synthetic and Endogenous TSPO Ligands and Their Binding Sites

### 5.1. Differences between PPIX and Synthetic Ligands in Interactions with TSPO

As stipulated at various points above, the synthetic TSPO ligands Ro5-4864, PK 11195, and FGIN-1-27 and the endogenous TSPO ligand PPIX have common but not identical effects. Historically, it was first established by displacement studies (typically applying [^3^H]PK 11195) that PPIX showed affinity for TSPO, as discussed above. A question that emerged from these studies was whether the binding sites on the TSPO for PPIX were identical or not. Subsequent studies dealt with this question and its ramifications by investigating which parts of the TSPO bound to its ligands. These studies are presented in this Section 5: “Structural relationships between synthetic and endogenous TSPO ligands and their binding sites”. These studies may also answer the question why these TSPO ligands affect similar functions, but in different ways (such apparent TSPO functions are discussed above).

In Figure 2, the structural differences between these TSPO ligands are shown. Regarding their binding sites on the TSPOs of various species, the structural relations with their TSPO ligand binding sites of PK 11195 (in mouse and *Bacillus cereus*) and PPIX (in *Rhodobacter sphaeroides*) were experimentally determined by crystallography and electron microscopy [9,69,99,100,101]. In a first study, Li *et al.* [99] expressed and purified the homologue of mammalian TSPO from *Rhodobacter sphaeroides* (*Rs*TSPO). They constructed a computational model of the *Rs*TSPO dimer using EM-Fold, Rosetta, and a cryo-electron microscopy density map. In this computational model it appeared that *Rs*TSPO binding sites for PK 11195 and PPIX were not identical, *i.e.*, not in the same location and without the same structure [9,99]. Furthermore, they reported that the equilibrium dissociation constant (Kd) for PK 11195 in *Rhodobacter sphaeroides* is 10 µM and for PPIX it is 0.3 µM [99], while for eukaryotes it is in the nM range [1,37]. Regarding mammals, Jaremko *et al.* [100] presented a three-dimensional high-resolution structure of mouse TSPO reconstituted in detergent micelles in complex with PK 11195. This mouse TSPO-PK 11195 structure is described by a tight bundle of TSPO’s five transmembrane α helices that form a hydrophobic pocket accepting PK 11195. The TSPO ligand binding site in question completely encapsulates PK 11195 [100]. As PPIX is larger than PK 11195, PPIX would not fit into this pocket. *Vice versa*, theoretically, by nature the larger binding pocket for PPIX would be large enough to accommodate the typical tricycle synthetic TSPO ligand. However, the location and structure of a mammalian PPIX binding site on TSPO has not been described. Interestingly, the mammalian gene for TSPO has 25 times more base pairs than the bacterial gene. Nonetheless, the bacterial and mammalian forms of TSPO by themselves present about the same number of amino acids and molecular weight (Table 1). Thus, one would expect that a PPIX binding site could be on the mammalian TSPO, even though its location and structure thus far have not been established. Thus, it is obvious that more studies dealing with structural ligand binding site interactions must be awaited to resolve such questions.

Studying *Rhodobacter sphaeroides*, Hinsen *et al.* [101] applied cryo-electron microscopy to tubular crystals of TSPO with lipids and analyzed possible ligand binding sites for protoporphyrin, PK 11195, and cholesterol, which appeared to not be identical, neither among each other nor with their mammalian counterparts. Guo *et al.* [69] reported on crystal structures for *Bacillus cereus* TSPO (*Bc*TSPO) down to a 1.7 Å resolution, including a complex with PK 11195. They also described *Bc*TSPO-mediated catalytic degradation of PPIX and showed that TSPO from *Bacillus cereus*, *Xenopus*, and human have similar PPIX-directed activities. In addition it was shown in *Rhodobacter sphaeroides* that TSPO appears to present a pocket that would be able to completely encapsulate an endogenous tetrapyrrole (porphyrin ligand) [9]. Keeping this in mind, it would be highly desirable to establish structural relations between PPIX and TSPO also in mammals, including explanations of the causes of binding competition between the endogenous PPIX and synthetic TSPO ligands. The recent studies on structural TSPO-ligand interactions provide very interesting data and interpretations. Nonetheless, it is also recognized that interpretation of the structure of a TSPO ligand binding site, including the interaction with the ligand, includes the bias of the observer [102]. In this respect, more studies are needed to address points of contention. As the list of extant TSPO ligands has become very long, it would be worthwhile to establish an effective computational approach to rigorously establish structural and functional commonalities and differences between all ligands, including their binding interactions with the TSPO.

Another essential question is how concentration-dependent bimodal effects of TSPO ligands come about. This is important because, for example, for drug development it is desirable that no adverse effects are induced at high doses, *i.e.*, accidental as well as intentional overdoses need to be precluded. In this context it was found that halogenation of the third, rotatable carbocycle of specific tricyclic TSPO ligands prevented lethal effects of otherwise identical ligands [22]. Also, moderation of the affinity of the ligands by specific modifications contributed to a reduction of adverse effects, while beneficial effects were promoted [22]. This may be important information for the selection of effective TSPO ligand–based drugs for treatments of various diseases.

### 5.2. Accommodation of Various Types of TSPO Ligands by Binding Sites on the TSPO

As mentioned, when looking at Figure 2, one can see that all tricyclic synthetic TSPO ligands shown have slightly different shapes. Regarding the tricyclic compounds, their ground form varies from hooked to straight, with acute angles, right angles, obtuse angles and straight angles at their connection points between the third carbocycle and the first two carbocycles. Furthermore, TSPO ligands can range from single carbocycle forms to multicarbocycle forms, including but not restricted to tetrapyrroles [7,46,103,104,105]. Looking at the PK 11195 binding pocket of mouse TSPO (as described by Jaremko *et al.* [101]), it would seem that none of these other TSPO ligands would comfortably fit into this PK 11195 binding site. This would, for example, be particularly true for the multicarbocycle forms of CB256 [92] and ZBD-2 [105], which are larger than PK 11195. Nonetheless, for example, ZBD-2 presents an affinity similar to PK 11195 [106].

One possibility is that the TSPO binding site can conform its shape to the particular shape of at least the tricyclic TSPO ligands. It is known that TSPO exchanges between multiple conformations in the absence of ligands [107], so it theoretically is possible that TSPO can conform its shape to various ligands. Alternatively, each ligand has a separate site on the TSPO. It is known that there are several binding sites for various molecules on the TSPO, including retinoic acid, curcumin, and a known Bcl-2 inhibitor, gossypol, apart from cholesterol, PPIX, and PK 11195, at least in *Rhodobacter sphaeroides* [99]. So the latter assumption that each ligand has its own docking site on the TSPO is also theoretically possible. In particular, bacterial studies regarding TSPO and its interactions with PPIX have provided us with titillating bits of information, but as of yet are not at a stage where they can solve all the questions regarding binding sites for TSPO ligands and interactions with tetrapyrroles including PPIX. Furthermore, it has been suggested that ligand binding sites on mammalian TSPO with high affinity for specific TSPO ligands are not presented by TSPO of all bacterial species [1]. More studies in prokaryotes as well as eukaryotes are needed to attain a better understanding of interactions between TSPO and its numerous binding agents.

In extensive studies, where the effects on affinity and function following small modifications of specific tricyclic cores, side chains, and halogenations of potential TSPO ligands were analyzed (as part of a project to design TSPO ligands with curative properties), it was extrapolated which components of TSPO ligands contributed to their affinity and which ones to function [21,22,90,91]. The side chain and the N atoms in the second carbocycle appeared to be important for affinity. The third carbocycle, including its halogenation, and also the N atoms in the second carbocycle were found to be important for function. We initially assumed that the first two carbocycles inserted into a binding pocket in the TSPO, while the third carbocycle would remain outside. In this way, the third carbocycle would present an element that could interact with the milieu surrounding the TSPO. The recent structural studies, however, show that while the first two carbocycles of PK 11195 indeed insert deep into a binding pocket in the TSPO, the remainder of the ligand is also encapsulated by the TSPO [9,69,99,100,101]. The question remains as to how the N atoms of the second carbocycle and the halogenation of the third carbocycle actually contribute to the functional characteristics of the ligands at hand. As PPIX also presents N atoms in its carbocycles and side chains, we assume that answers to the questions may also be relevant for understanding PPIX-TSPO interactions and their effects.

The observations of various binding sites for various factors on the TSPO may present some explanation for the context-dependent responses of TSPO to various stimuli. While this on the one hand defines the complexity of TSPO characteristics, making it enigmatic for our understanding [1], on the other hand it exemplifies TSPO’s functional versatility against the various life-threatening challenges for animals as well as plants and bacteria [7]. This evolutionarily conserved protein and its endogenous ligands, including tetrapyrroles such as PPIX, allow organisms from prokaryotes to eukaryotes to respond not just to toxic environmental insults presented by chemicals to unicellar organisms, but also to challenges to multicellular organisms, even to physical and mental injuries emanating from human social interactions (ranging from inflammation and traumatic brain injury to maladaptive responses to stress, including anxiety).

## 6. Bacteria, TSPO, and Tetrapyrroles

**Figure 3:**
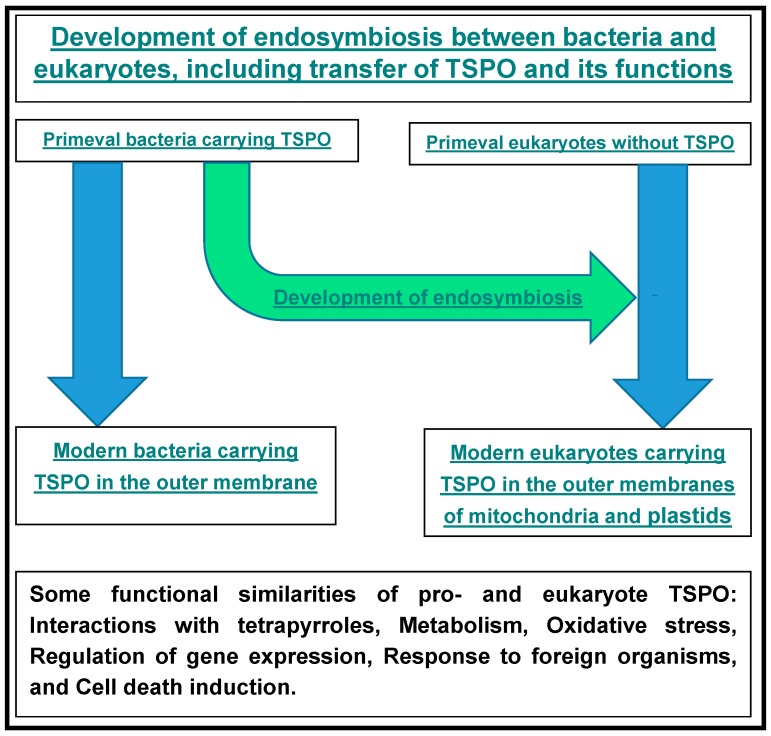
This scheme very concisely summarizes evolutionary relations between bacteria and eukaryotes regarding the presence of TSPO in these life forms, as well as the associated functions. In particular, it appears that during evolution, originally free living bacteria with TSPO became part of eukaryotes in the form of organelles, such as mitochondria and plastids with TSPO. In eukaryotes the TSPO functions that can be found in bacteria appear to basically have been maintained in cellular organelles. Beyond this, in prokaryotes as well eukaryotes, including multicellular organisms, TSPO serves to maintain homeostasis and viability.

TSPO has also been identified in the genome of bacteria (Table 1). As reviewed by Li *et al.* [99], TSPO was discovered in the carotenoid gene cluster known as CrtK in purple non-sulfur bacteria [108]. Purple non-sulfur bacteria are considered to be closely related to the free living bacterial ancestors of mitochondria [109]. TSPO was discovered first in *Rhodobacter capsulatus* and *Rhodobacter sphaeroides* [108,110,111]. The acronym TSPO is originally derived from the name tryptophan-rich sensory protein (TspO) which was first applied to these bacteria [4,110,111]. In 2009, Chapalain *et al.* [112] identified 98 bacteria presenting TSPO in their genome. Bacterial TSPO typically appears to be organized as a dimer, and in general the affinity for PK 11195 is in the same nM range as for eukaryotic TSPO. Treating *Pseudomonas fluorescens* MF37 with PK 11195 (10^−5^ M) increased adhesion to living or artificial surfaces and biofilm formation activity; at the same time, the apoptotic potential of bacteria on eukaryotic cells was significantly reduced [112]. As several structural and functional characteristics are shared with the mammalian TSPO, they may present an original source of TSPO function (see also Table 2). It appears that sometime during evolution, relationships between eukaryotes and particular prokaryotes changed from lethal to symbiotic (Figure 1 and Figure 3). For example, eukaryotes ingesting prokaryotes did not digest them or prokaryotes originally clinging to eukaryotes and inducing programmed cell death become internalized endosymbionts and typically no longer induce programmed cell death. Assuming that PPIX and TSPO coexisted at the time point of this evolutionary transition, they may have been important participants in the establishment of this endosymbiotic relationship between bacteria and eukaryotes.

As TSPO of Archeobacteria is homologous to TSPO of species from the other kingdoms (Table 1), TSPO indeed is an evolutionarily conserved protein [1,2]. Moreover, rat TSPO has been shown to substitute for TSPO in *Rhodobacter sphaeroides* (*Rs*TSPO), also indicating conserved functional characteristics [113]. Similarly, PPIX is also present from archea to eukaryotes [114]. It has been recognized that the location of TSPO in bacteria and eukaryotes is similar. As in mitochondria, in bacteria including *Rhodobacter* TSPO is located in the outer membrane [113,115]. *Rhodobacter* TSPO is involved in regulating photosynthetic gene expression in response to oxygen and light conditions [113,115]. It has been suggested that TSPO-porphyrin interactions underlie the regulation of this gene expression, allowing *Rhodobacter* to switch from oxygen respiration to photosynthesis and back [116]. A question is whether tetrapyrroles act on TSPO as ligands to induce TSPO to activate its function as a nuclear gene expression regulator, or whether tetrapyrroles present intermediate steps for the mechanisms whereby TSPO regulates nuclear gene expression. Similar to its mammalian and plant orthologs, in *Rhodobacter sphaeroides* TSPO appears to be involved in the transport of small molecules such as porphyrin intermediates of the heme and in chlorophyll biosynthesis-degradation pathways [115,117]. *Rs*TSPO shares considerable sequence homology with human TSPO (*Hs*TSPO). Apart from an overall significant level of sequence identity (30%), *Rs*TSPO presents particular sequence similarity with *Hs*TSPO in the first extra-membrane loop (loop 1) considered to participate in porphyrin binding [117], synthetic TSPO ligand binding [118], and cholesterol binding [119]. Regarding *Rs*TSPO, it has been suggested that PK 11195 and PPIX interact with different sets of tryptophans [99]. Furthermore, the Kd in *Rhodobacter sphaeroides* for PK 11195 is 10 µM and for PPIX it is 0.3 µM, *i.e.*, not the same as for eukaryotes, where these Kds are in the nM range [99]. *E. coli* does not even present appreciable TSPO ligand binding [1]. Nonetheless, as mentioned above, Kd for TSPO in bacteria typically appears to be in the nM range [112]. As discussed above, high affinity and low affinity TSPO ligand binding sites can also be found in mammals [10,28,60,62,63,64,65,66,67]. Thus, physiological functions of TSPO and tetrapyrroles in bacteria are reminiscent of those in animals (see also Table 2). It indeed is tempting to correlate this with the bacterial origin of mitochondria, including their TSPO [99] (see also Section 8 dealing with *“*TSPO-tetrapyrrole interactions from an evolutionary perspective*”*).

## 7. Plants, TSPO, and Tetrapyrroles

Plant TSPO homologs, with a molecular size of 18–20 kDa, show 15%–22% identical residues with bacterial and mammalian TSPO [120]. Interestingly, the various plant TSPO homologs present a distal segment of 40–50 amino acids, which is not identical to the bacterial and mammalian counterparts [120,121]. Similar to the mammalian and bacterial TSPO, plant TSPO homologs have a high affinity binding site, and a second binding site with a low affinity (µM range) has also been reported [120]. Several endogenous TSPO ligands were found in plants, such as benzodiazepines (including delorazepam and temazepam), porphyrins, cholesterol, and diazepam binding inhibitor (DBI) [120]. Regarding animal TSPO research, no endogenous, diazepam-like TSPO ligands have been identified in animals to date. TSPO cellular localization in plants can be in the mitochondria and plastids, as well as in the nuclear fraction, the endoplasmatic reticulum, and the Golgi stacks [120,122,123]. These various localizations may relate to TSPO’s function in the translocation of tetrapyrrole intermediates across organelle membranes [124]. In this context, it was shown, both *in vitro* and *in vivo*, that the plant TSPO is able to bind heme and PPIX [125].

Four major tetrapyrroles are biosynthesized in the plastids of higher plants: chlorophyll, heme, siroheme, and phytochromobilin [124]. Among the plant tetrapyrroles, chlorophyll and siroheme act in plastids, heme is universally distributed to all cellular compartments, and phytochromobiline occurs in the cytoplasm [47,126]. Regarding *Arabidopsis thaliana* TSPO (*At*TSPO), several genes related to tetrapyrrole biosynthesis were downregulated in an *At*TSPO knockdown cell line, indicative of TSPO regulation of the tetrapyrrole metabolism [124]. *Vice versa*, treatment of the wild-type plants with tetrapyrrole biosynthesis inhibitors increased TSPO mRNA levels. Furthermore, mutations in different genes for tetrapyrrole metabolism also affect TSPO expression levels. For example, mutations in *gun4* (protoporphyrin IX- and Mg-Protoporphyrin IX-binding protein) lead to increased TSPO levels [124]. *At*TSPO levels are regulated at the transcriptional, post-transcriptional and post-translational levels in response to abiotic stress conditions [124]. Feeding the PPIX precursor 5-aminolevulinic acid (ALA) to *Arabidopsis thaliana* seeds enhanced porphyrin biosynthesis as well as downregulation of AtTSPO, and improved salt tolerance [123,124]. Heme synthesis was suggested to be responsible for TSPO downregulation [125]. Treatment of *Arabidopsis* cell culture with inhibitors of porphyrin biosynthesis significantly increased *At*TSPO expression, compared to the control [125].

It was proposed that TSPO serves to protect seed germination from the toxic effects of tetrapyrroles [123]. Plant TSPO is able to bind heme, as for example is required for TSPO degradation through autophagy [125,126]. In an *Arabidopsis* transgenic cell line over-expressing TSPO, ROS levels were found to be higher than in the wild type. This suggests that the plant TSPO, which is considered a heme scavenger, may mediate ROS homeostasis [127]. Moreover, TSPO levels are decreased 48 h after abscisic acid (ABA)-induced stress [125]. It is possible that heme levels are regulated by the over-expressed TSPO during ABA stress [128]. Further evidence for TSPO-tetrapyrrole interactions related to stress came from studies on the moss *Physcomitrella patens*, which has three TSPO homologs [129]. Under stress conditions, the *Pp*TSPO1 null mutants show elevated H_2_O_2_ levels, enhanced lipid peroxidation and cell death, indicating an important role of *Pp*TSPO1 in redox homeostasis. Furthermore, in *Physcomitrella patens*, knockout of one of its TSPOs led to increased levels of heme and PPIX [129]. These knockdown mutants had higher activity of class III peroxidase (PRX34), which is produced as a defense response to pathogens and is responsible for the oxidative burst response. Over-expression of other oxidative stress-related genes was seen in these mutants [130]. Overall, it appears that stress reduction leads to decreased levels of TSPO. These various studies in plants suggest that, possibly, increased levels of TSPO serve to reduce the stress response, which can be prevented by TSPO knockout and TSPO knockdown.

Furthermore, TSPO expression appears to be related to plant development. For example, dry seeds have high levels of TSPO. In contrast, TSPO levels in plantlets and leaves are undetectable [121]. This is reminiscent of neurodevelopment observed for mouse cells, where TSPO is abundant in neural progenitor cells but not detected in the derived healthy, mature neurons [32]. Tetrapyrroles also may be inseparable from development. For example, siroheme deficiency affects plant growth and development [131], reminiscent of PPIX promoting migration of neurons in embryonic insects [84].

Thus, physiological functions of TSPO in plants, including its interactions with tetrapyrroles, appear to be comparable to what is observed in animals and bacteria. Such functions range from embryonic development to the stress response in adult life (see also Table 2). Obviously, more studies in plants regarding TSPO-tetrapyrrole interactions, by themselves and in comparison to such interactions in animals and bacteria, will give very interesting insights in the life-supporting functions of TSPO. This will have implications in future approaches to human health issues, as well as agricultural production, including plant cultivation and animal breeding. Such research comparing bacterial, plant, and animal TSPO characteristics will also have implications for our understanding of TSPO evolution, including its interactions with tetrapyrroles.

## 8. TSPO-Tetrapyrrole Interactions from an Evolutionary Perspective

As valid for animal mitochondria endosymbiosis, plastids and mitochondria of plants also derive from prokaryotic symbionts [132,133]. Without wanting to go too much into detail of what is known regarding the organelle evolution of eukaryotes, we mention here that plants present the oldest eukaryote fossils. Microfossils of algae have been found in *ca.* 1.5-billion-year-old rocks in northern Australia [132,133]. Moreover, steroid molecules preserved as steranes have been identified in samples of sediments from 2.5 to 2.8 billion years old [134]. Thus, these studies suggest that eukaryotic algae already existed during the Late Archean period, implying that the endosymbiotic relation of eukaryotes and prokaryotes already had been established at that time. Interesting for our perspective from TSPO research, in particular regarding TSPO functions associated with steroidogenesis, is the early occurrence of steroid production in the geological record. It has been appreciated for some time that evolutionary aspects of TSPO can have implications for our understanding of TSPO function [1,135]. Regarding prokaryotes, present day cyanobacteria possess TSPOs that appear to be associated with stress, ROS generation, cell cycle regulation, heme metabolism, and homeostasis [136,137,138], reminiscent of TSPO functions in eukaryotes (see also Table 2, Figure 1 and Figure 3). It appears that iron ores have been deposited by cyanobacteria already around four billion years ago, as a consequence of typical metabolic characteristics of cyanobacteria [139]. So, even as no direct evidence is available, it can be suggested that TSPO has been present for about four billion years in the evolution of Earth’s living organisms [139,140].

These studies, suggesting the early existence of the bacterial ancestors of the endosymbiotic mitochondria and plastids, allow for the assumption that eukaryote evolution, including endosymbiosis of mitochondria and plastids with their TSPO, may indeed have been initiated early in the Earth’s existence. The same appears to be true for PPIX. For example, the phylogenetic distribution of enzymes for the tetrapyrrole biosynthesis pathway, as described by Kobayashi *et al.* [141], parallels well-acknowledged views of evolution on mitochondrial and plastid endosymbiosis [132,133]. Briefly, in the tetrapyrrole biosynthesis pathway, protoporphyrinogen IX oxidase (Protox), present in mitochondria and plastids, catalyzes the formation of PPIX, the last common intermediate for the biosynthesis of heme and chlorophyll. In particular the phylogenetic distribution of HemY, one of the three nonhomologous isofunctional Protox forms (HemG, HemJ, and HemY), reflects endosymbiosis with bacteria in the evolution of eukaryotes. HemY is ubiquitous in prokaryotes, including cyanobacteria and purple bacteria, and is the only Protox in eukaryotes. These studies [132,133,141] suggest that HemY may be the ancestral bacterial Protox form nowadays present in the mitochondria and plastids of eukaryotes. Furthermore, regarding genes for the synthesis of bacteriochlorophyll from PPIX, by phylogenetic analysis of available genomic data it can be postulated that the transfer of such genes took place from cyanobacteria to purple non-sulfur phototropic bacteria *(Rhodobacter* and *Rhodopseudomonas*), at least as early as the Proterozoic era [142], which ranges from 2500 to 542.0 ± 1.0 million years ago, presenting in the most recent part of the Precambrian. Also from a phylogenetic perspective, studies on *Rhodobacter sphaeroides* have suggested that TSPO’s association with larger membrane channels, such as VDAC in eukaryotes and porin in prokaryotes, can be considered a conserved TSPO characteristic [140]. This is particularly so as purple non-sulfur phototropic bacteria are considered the free living ancestral form of mitochondria [109].

Thus, we see the emergence of a body of data indicating that vital functions of TSPO such as modulation of membrane potential, steroidogenesis, and tetrapyrrole synthesis appear to already have been present very early in the geological history. As in present day organisms, these functions are unequivocally associated with the TSPO in bacteria, as well as in the mitochondria and plastids of eukaryotes, and it can be assumed that TSPO was present early on in the geological history of the Earth. A time point of 3.5 billion years ago has been postulated [140]. The same can be said for the existence of tetrapyrroles such as PPIX. Thus, TSPO-tetrapyrrole interactions indeed may have originated very early on in the evolution of living organisms.

## 9. Conclusions and Perspectives

In conclusion, TSPO and tetrapyrroles including PPIX are universally distributed, apparently in all six kingdoms of living organisms. Importantly, PPIX and TSPO demonstrate structural and functional interactions. Their life-supporting functions are of a wide variety, including gene expression, membrane functions, and programmed cell death, that translate to the regulation of homeostasis including adequate responses to environmental challenges. In this context, TSPO can be considered a receptor for PPIX, a transporter for tetrapyrroles, and a participant in the regulation of tetrapyrrole metabolism; *vice versa*, tetrapyrroles can modulate TSPO functions. Figure 3 presents a schematic overview of the evolution of endosymbiosis, from free living bacteria to mitochondria and plastids in animals and plants, including associations with TSPO functions.

Within this perspective it appears that further research, basic as well as applied, may give relevant information on how these functional relations between tetrapyrroles and TSPO can give insights into various biological mechanisms relevant for human health issues as well as agricultural advances. As TSPO-tetrapyrrole interactions appear to relate to eukaryote-prokaryote endosymbiotic relations (Figure 1), such research may also give insights into basic biology questions ranging from evolution to ecology. A better understanding of structure-function interactions between TSPO and its endogenous ligands such as tetrapyrroles, including PPIX, may aid in the development of new synthetic TSPO ligands as adequate drugs for treatments of various diseases. Some basic elements for such structural characteristics are indicated in Figure 2. It is noteworthy that diseases that have been associated with TSPO and its functions present a very broad range, for example including but not restricted to: porphyrias, developmental disorders, inflammatory diseases, cancer, and neuropathological disorders, including maladaptive responses to stressors. Finally, the application of TSPO ligands with efficacious curative effects may enhance the life span of patients as well as healthy individuals.

## Figures and Tables

**Table 1:** Translocator protein (TSPO) gene length in base pairs (bp) and protein length in amino acids (aa) in different species that are discussed in this review, plus a few additional ones, to obtain a representative view of what is known regarding TSPO in living organisms in general. The left column gives the names of the species. The species are organized according to: human, mammals, insects, archea, bacteria, plants, and fungi. The middle column shows the TSPO gene lengths in base pairs (bp) for each species. The right column shows the protein lengths, which are between 151 and 211 aa. TSPO protein molecular weight for all species typically is 18 kDa. Interestingly, while protein size does not differ essentially from species to species, as shown here, gene length varies from 11,729 bp in humans to as low as 456 bp in *Bacillus anthracis str. Ames*. (After the resources “Gene” and “Protein” from the National Center for Biotechnology Information, National Library of Medicine, 8600 Rockville Pike, Bethesda, MD, USA).

Various Species Expressing TSPO	TSPO Gene Length (bp)	TSPO Protein Length (aa)
*Human*		
*Homo sapiens*	11,729 bp	169 aa
*Mammals*		
*Rattus norvegicus*	10,253 bp	169 aa
*Mus musculus*	10,631 bp	169 aa
*Amphibians*		
*Xenopus tropicalis*	5970 bp	211 aa
*Insects*		
*Drosophila melanogaster*	6569 bp	185 aa
*Aedes aegypti*	1236 bp	176 aa
*Archea*		
*Haloferax mediterranei*	486 bp	161 aa
*Bacteria*		
*Bacillus anthracis str. Ames*	456 bp	** 151 aa
*Rhodobacter sphaeroides*	480 bp	158 aa
*Rhodobacter capsulatus*	483 bp	** 160 aa
*Plants*		
*Arabidopsis thaliana*	1044 bp	196 aa
*Solanum tuberosum*	895 bp	203 aa
*Ricinus communis*	1530 bp	196 aa
*Vitis vinifera*	1055 bp	185 aa
*Fungi*		
*Cryptococcus gattii*	865 bp	174 aa
*Schizosaccharomyces cryophilus*	480 bp	164 aa
*Aspergillus fumigatus*	632 bp	177 aa
*Kluyveromyces*	486 bp	160 aa

**Table 2:** TSPO is involved in various functions in animals, plants, and bacteria. As described in this review, and summarized in this table, TSPO performs, regulates, and modulates a rich spectrum of life essential functions. These TSPO functions have been studied extensively in mammals, ranging from molecular biological mechanisms to various stress responses, at cellular and organismal levels, and even to enhancement of life expectancy. (Note: TSPO functions uncovered in insects show great similarity to those in mammals.) While research on plant and bacterial TSPO thus far has been more restricted, the TSPO functions described in various plant and bacteria species are reminiscent of several TSPO functions described in animals. In this table, comparable functions of animals, plants and bacteria are placed in one row.

TSPO-Associated Functions in Animals, Plants, and Bacteria		
Animals	Plants	Bacteria
Mitochondrial membrane potential transition		Interactions with large membrane channels
Transport of porphyrin intermediates	Translocation of tetrapyrrole intermediates	Transport of porphyrin intermediates
Heme metabolism	Tetrapyrrole metabolism	Heme metabolism
ROS generation	Oxidative stress	ROS generation
Programmed cell death	Cell death	Induction of apoptosis in eukaryotes
Mitochondrial protein transport		
Mitochondrial metabolism		Anaerobic and aerobic metabolism
Mitochondrial cholesterol transport		Cholesterol binding
Steroidogenesis		
Nuclear gene expression		Gene expression
Cell cycle	Cell cycle	Cell cycle
Cell growth		Cell growth
Cell proliferation		
Cell migration		
Cell adhesion		Adhesion
Cell differentiation		
Embryonic development	Seed and plant development	
Endocrinological function		
Reproduction		
Stress response	Stress response	Stress response
Immune response	Response to pathogens	
Inflammatory response		
Glial activation		
Response to brain disease and injury		
Emotional health		
Mental health		
Cardiovascular health		
Homeostasis	Homeostasis	Homeostasis
Life span of multicellular organisms		

**Table 3:** TSPO functions affected by tetrapyrroles in animals, plants and bacteria. While it is known that tetrapyrroles can bind TSPO in animals, plants, and bacteria, TSPO functions affected by porphyrins have been described in particular for animals and humans. Although not studied as extensively as in animals and humans, tetrapyrrole effects on TSPO functions in plants and bacteria are reminiscent of those described for animals and humans.

TSPO Functions Affected by Tetrapyrroles		
Animals	Plants	Bacteria
TSPO expression	TSPO expression	
Mitochondrial membrane potential transition		
ROS generation	Stress response	
Mitochondrial protein transport		
Mitochondrial cholesterol transport		
Regulation of steroidogenesis		
Heme metabolism		
Transport of porphyrin intermediates		
Modulation of nuclear gene expression	Seed and plant development	Photosynthetic gene expression
Cell migration		
Programmed cell death		
Mitochondrial metabolism		Switch between anaerobic and aerobic metabolism
Life span		
